# Authors’ Reply: Citation Accuracy Challenges Posed by Large Language Models

**DOI:** 10.2196/73698

**Published:** 2025-04-02

**Authors:** Mohamad-Hani Temsah, Ayman Al-Eyadhy, Amr Jamal, Khalid Alhasan, Khalid H Malki

**Affiliations:** 1 Pediatric Department, College of Medicine, King Saud University, King Abdullah Road, Riyadh, 11424, Saudi Arabia, 966 114692002; 2 Department of Family and Community Medicine, King Saud University Medical City, Riyadh, Saudi Arabia; 3 Research Chair of Voice, Swallowing, and Communication Disorders, Department of Otolaryngology-Head and Neck Surgery, College of Medicine, King Saud University, Riyadh, Saudi Arabia

**Keywords:** ChatGPT, Gemini, DeepSeek, medical education, AI, artificial intelligence, Saudi Arabia, perceptions, medical students, faculty, LLM, chatbot, qualitative study, thematic analysis, satisfaction, RAG retrieval-augmented generation

 We appreciate the thoughtful critique of our manuscript “Perceptions and earliest experiences of medical students and faculty with ChatGPT in medical education: qualitative study*”* [1] by Zhao and Zhang [2]. Concerns over the generation of hallucinated citations by large language models (LLMs), such as OpenAI’s ChatGPT, Google’s Gemini, and Hangzhou’s DeepSeek, warrant exploring advanced and novel methodologies to ensure citation accuracy and overall output integrity [3].

The LLMs have demonstrated a propensity to generate well‐formatted yet fictitious references—a limitation largely attributed to restricted access to subscription-based databases and their reliance on probabilistic text generation [4]. As LLMs evolve, future iterations may integrate more reliable retrieval-based architectures, enhancing their capacity to cite legitimate sources while reducing fabricated references [4,5]. However, until such improvements are systematically validated, scholars must remain cautious.

One suggested enhancement is using retrieval-augmented generation (RAG) [6]. This approach integrates up-to-date external information, substantially improving real-world applicability. However, even RAG-based systems can misinterpret or distort source content under high-trust conditions. To address this, the authors developed Hallucination-Aware Tuning (HAT) [6]. HAT trains dedicated detection models to generate labels and detailed descriptions of identified hallucinations. These descriptions are then used by GPT-4 to correct discrepancies. The combination of corrected and original outputs forms a preference dataset that, when used for Direct Preference Optimization training, yields LLMs with reduced hallucination rates and improved answer quality [6].

We also propose another solution aimed at fundamentally reducing citation errors: the development of “Reference-Accurate” academic LLM by major global publishers. Leading journals could develop their own specialized LLM, trained exclusively on rigorously verified academic literature from robust databases. This targeted training would ensure that every generated reference is accurate and directly traceable to published work. Ideally, these publisher-backed LLMs would be made freely available to promote open science.

Therefore, we recommend a dual approach that combines advanced RAG methodologies with publisher-developed academic LLMs. Comparative studies should be conducted to evaluate the citation accuracy, factual consistency, and overall performance of RAG-HAT-tuned models against these publisher-specific models. Collaborative efforts among academic institutions, publishers, and AI developers are essential to establish standardized protocols and reliable training datasets. Such partnerships would not only enhance the reliability of LLM-generated outputs but also foster greater trust in AI-assisted scholarly communication.

Moreover, the broader academic community bears responsibility for critically appraising AI-generated content. While LLMs can streamline information retrieval and synthesis, human oversight remains indispensable for safeguarding academic integrity. Rather than dismissing AI-driven tools due to their current flaws, we advocate for further research to ensure greater alignment with evidence-based scholarship and authentic publications. Future LLM iterations may rapidly overcome these limitations, but until then, transparency, responsible usage, and ongoing improvements in AI training remain imperative.

In conclusion, while RAG augmented by HAT represents a potential advancement in reducing hallucinations, the development of specialized, reference-accurate academic LLMs by publishers may offer a promising pathway. By integrating both strategies and ensuring human oversight, the academic community can ensure that AI-driven tools reliably support the rigor and transparency essential to scholarly research.

## Abbreviations

HAT: Hallucination-Aware Tuning
LLM: large language model
RAG: retrieval-augmented generation
